# Understanding Public Knowledge, Attitudes, and Awareness Toward Seizure Episodes Among Residents of Madinah: A Saudi Cross-Sectional Study

**DOI:** 10.1155/bn/2142400

**Published:** 2025-02-15

**Authors:** Bayan M. Almarwani, Sadin Ayman Alamri, Aseel Ayman Alamri, Ahmad Salim Badawi, Zakaria Yahya Khawaji, Afnan Mohammad Akhwan, Basel MohammedBassam Garah, Abdullah A. Tawakul

**Affiliations:** ^1^Department of Medicine, Taibah University, Madinah, Saudi Arabia; ^2^Collage of Medicine and Surgery, University of Jeddah, Jeddah, Saudi Arabia; ^3^Collage of Medicine and Surgery, Taibah University, Madinah, Saudi Arabia; ^4^Department of Medicine, Faculty of Medicine, Umm Al-Qura University, Makkah, Saudi Arabia

**Keywords:** attitude, awareness, epilepsy, knowledge, Saudi Arabia, seizure

## Abstract

**Background:** Epilepsy is a prevalent condition that affects a large population. However, a lack of knowledge and misconceptions about seizures can lead to inappropriate reactions and negative attitudes toward people with epilepsy, who are more likely to experience stigma because of their illness. This study explored public knowledge, attitudes, and awareness of epilepsy and seizures among residents of Madinah, Saudi Arabia.

**Methods:** A descriptive, cross-sectional study was conducted between September and November 2023 involving 2626 random adult residents of the Madinah Region in Saudi Arabia. Data were collected through an online survey consisting of 27 questions concerning epilepsy.

**Results:** Regarding participants' awareness of epilepsy, 92.2% reported having heard or read about it, and 12.8% had attended a course on controlling epileptic seizures. The results showed an acceptable level of understanding about epilepsy. The mean knowledge and attitude scores about epilepsy for the sample were 22 (SD ± 3.2) and 27.1 (SD ± 3.1), respectively, indicating moderate knowledge and positive attitudes toward epilepsy. Among demographic characteristics, knowledge about epilepsy was significantly associated with gender, age, marital status, educational level, occupation, household monthly income, having heard about epilepsy, having a family member diagnosed with epilepsy, and course attendance. However, attitudes toward epilepsy were only significantly associated with gender, having heard about epilepsy, having a family member diagnosed with epilepsy, and course attendance. Similarly, actions toward seizure attacks were significantly associated with all demographic characteristics except education.

**Conclusions:** The residents of Madinah have generally positive attitudes regarding epilepsy, although their knowledge levels could be enhanced by education. Therefore, more educational awareness campaigns are required to improve public understanding of epilepsy and appropriate actions to take when witnessing a seizure. The results of this study provide a foundation for worldwide comparisons of general levels of knowledge and attitudes concerning epilepsy in Saudi Arabia.

## 1. Introduction

Epilepsy is one of the most prevalent neurological disorders, affecting 50 million individuals globally, according to the World Health Organization (WHO) [1]. Epilepsy is characterized by a chronic tendency to have repeated seizures that are not associated with an explicit immediate cause. Seizures are defined as transient signs and symptoms of abnormal excessive synchronous neuronal firing in a region of the brain or across the entire brain [2]. Seizure symptoms vary widely. They can manifest as brief motor or nonmotor (altered sensation, cognition, or mood) conditions with or without altered awareness [3, 4]. The most common causes of seizures in children are genetic factors, perinatal injuries, and cortical developmental abnormalities, while encephalitis/meningitis, traumatic brain injury, and brain tumors are major causes of seizures in older individuals without a genetic predisposition to the condition [1, 5, 6].

The prevalence of epilepsy in Saudi Arabia has been estimated to be 6.54/1000 individuals, which is similar to the rates reported in other locations [7]. According to the WHO, the estimated prevalence of active epilepsy in the general population at a given time is between four and 10 per 1000 people [1]. People with epilepsy (PWE) are likely to experience stigma because of their illness, leading to emotional and psychological consequences. Studies have shown that PWE have a higher prevalence of major depressive syndrome and anxiety, along with a decreased quality of life due to discrimination and negative attitudes [8, 9]. According to previous research, there is a lack of public awareness in Saudi Arabia regarding what causes seizures and how to respond when witnessing a seizure [10]. Moreover, negative attitudes toward PWE are commonly reported, highlighting the importance of public education and advancement in seizure knowledge [10–13]. Our research explored societal awareness of seizures among residents of Madinah to assess the need for educational initiatives and campaigns.

In 2022, research was conducted in Makkah City to assess public knowledge and awareness of seizure attacks. Of the 401 participants, 32.9% reported a good knowledge level versus 67.1% reporting a poor level [14]. In a study of 448 residents of the Eastern Province of Saudi Arabia, 24.8% of the sample reported a good level of knowledge about epilepsy, and males and females had similarly positive attitudes toward PWE (50.3% and 43.1%, respectively) [15]. In Rabigh Province, 87% of 511 respondents reported they were unsure of what to do if someone was having an active seizure, beyond removing them from danger [16]. A systematic review and meta-analysis of 27 studies in 2021 reported that people's overall awareness level was unsatisfactory and that the population's attitude regarding marriage, employment, and children was negative [10].

In 2019, Alsohibani et al. conducted a study with 3800 individuals in Al-Qassim, Saudi Arabia. The data revealed that 73.2% of parents would permit their children to play with a peer with epilepsy, 35.7% would allow their children to marry someone with epilepsy, and 74.9% believed that PWE are equally capable of fulfilling job responsibilities [13]. Another study conducted in Jeddah with 1940 participants reported that 17.1% of respondents thought epilepsy was the result of a genetic disorder, while 60% believed it is a brain disease. In terms of management, 50% of the participants thought epilepsy could be treated by medications, and 31.5% believed that recitation of the Quran could be a treatment for this disease [17]. A cross-sectional study conducted in local communities in Saudi Arabia found that 24.5% of male respondents and 26.2% of female respondents believed PWE should be allowed to drive [18].

Seizure episodes are a common neurological condition that affects a significant portion of the population. However, a lack of public understanding and misconceptions surrounding seizure episodes have been observed. These misconceptions can result in inappropriate responses when individuals witness a seizure episode. This study investigated public awareness, knowledge, and attitudes toward seizure episodes to gather valuable insights, identify areas where misinformation exists, and determine the need for public education. Additionally, the data provide insights into the effectiveness of current first aid and educational programs. By addressing these aims and objectives, we aimed to enhance public awareness and foster appropriate responses, leading to improved outcomes for individuals experiencing seizure episodes.

## 2. Research Objectives

### 2.1. Primary Objective

The primary objective of this study is to assess the public's knowledge, awareness, and attitudes toward seizure episodes.

### 2.2. Secondary Objectives

The secondary objective of this study is to investigate the behaviors of the public when witnessing a seizure episode.

## 3. Methodology

### 3.1. Study Design

This descriptive, questionnaire-based cross-sectional study was conducted between September and November 2023 with a sample of adults from Madinah City, Saudi Arabia.

### 3.2. Study Population

The study population included adults at least 18 years of age from Madinah City, Saudi Arabia. Individuals less than 18 years of age or who do not live in Madinah were excluded.

### 3.3. Sampling Method

The researcher used a convenience sampling approach to collect data from a conveniently available pool of respondents. People living in Madinah City were easily accessible to the researcher and were thus surveyed in this study. All participants were asked of the city they were living.

### 3.4. Study Sample

The essential sample size was estimated using Raosoft online software (https://www.raosoft.com/samplesize.html). The predicted 50.0% prevalence of epilepsy awareness, epilepsy knowledge, and epilepsy first aid; a 95% confidence level; and a margin of error of 5% were used in the calculation. With an estimated population of 1,570,000 in Madinah, the required sample size was determined to be 385. Fifty volunteers from Madinah facilitated the recruitment of participants by disseminating the online survey to all their connections. For data analysis, a response rate of 60% or above was desired. To prevent duplicate responses from single participants, email addresses were obtained from all participants, and those with the same email address were prevented from completing the questionnaire more than once. The data were also screened for duplicate entries and/or participants having the same email address before the data analysis.

### 3.5. Consent and Ethical Approval

Before engaging in this study, all participants provided informed consent. They were presented with a thorough online consent form detailing the study's purpose and outlining confidentiality measures. After reviewing the form, participants had the option to indicate their voluntary agreement to participate by selecting the “I Agree” checkbox. Alternatively, they could choose the “I Do Not Agree” option if they decided not to participate. Importantly, no participant names or identifying information were collected during the study. The data were stored in software that is not prone to being breached by unauthorized persons. The study was conducted in line with the Helsinki protocol, and ethical approval was acquired from the Institutional Review Board of Umm Al-Qura University, Makkah, before beginning the study (HAPO-02-K-012-2023-09-1758, dated September 27, 2023).

### 3.6. Research Instrument

Each participant received an online link to a self-administered questionnaire. The survey employed in this study was adapted from a literature review of previous pertinent studies conducted in several countries [14, 15, 19–22]. In these studies, the scale underwent development, validation, and translation into Arabic. The instrument contained structured questions divided into five sections: (a) demographic information, including gender, age, marital status, educational level, occupation, and family monthly income; (b) awareness of epilepsy (5 items); (c) knowledge about epilepsy (5 items); (d) attitudes toward epilepsy (10 items); (e) and actions while witnessing seizures (7 items). All questions regarding awareness and knowledge about epilepsy and actions in response to seizure attacks were measured on a yes/no scale, with “*yes*” coded as 1 and “*no*” coded as 0. Similarly, questions on attitudes toward epilepsy were measured on three-point Likert scale, ranging from *disagree* (1) to *agree* (3). The scores on each question were summed to calculate the respective latent variables, which were then categorized into two groups: (i) poor knowledge (scores between 10 and 21) and good knowledge (scores between 22 and 31); (ii) negative attitude (scores between 10 and 27) and positive attitude (scores between 28 and 30); and (iii) bad actions (scores between 0 and 3) and good actions (scores between 4 and 6). The survey was conducted online using Google Forms.

### 3.7. Data and Statistical Analysis

The data for this study were analyzed using the Statistical Package for Social Sciences software, version 26.0 (SPSS Inc., Chicago, Illinois). Categorical variables and participants' responses were presented as frequencies and percentages. The association between knowledge, attitude, and practice toward epilepsy and participants' sociodemographic characteristics was examined using the chi-square test for categorical variables. A *p* value of less than 0.05 was considered statistically significant, and the confidence interval was 95%.

## 4. Results

After excluding 46 individuals who did not agree to participate in the study, the sample consisted of 2626 participants residing in Madinah with a response rate of 98.3%.  Table 1 presents the sociodemographic characteristics of the sample. Of the participants, 32.9% identified as male, and 67.1% identified as female. Most participants (65.7%) fell within the age range of 18–30 years, and 50.3% of them were students. Additionally, 63.7% of the sample were single. Regarding education, most participants (61.8%) had completed a bachelor's degree.

**Figure 1:**
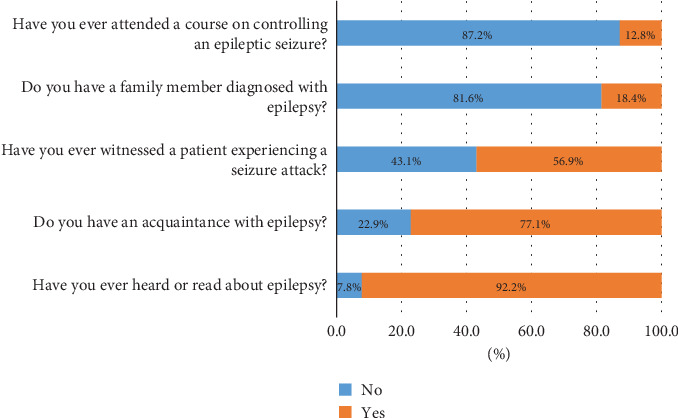
Distribution of respondents across awareness about epilepsy.

Figure 1 portrays the participants' awareness of epilepsy. Most participants (92.2%) reported having heard or read about epilepsy, and 77.1% indicated having a personal acquaintance who has epilepsy. In contrast, a small portion (18.4%) of the sample had a family member diagnosed with epilepsy, and 12.8% had attended a course on controlling epileptic seizures. However, over half of the participants (56.9%) reported having witnessed someone experiencing a seizure.

**Figure 2:**
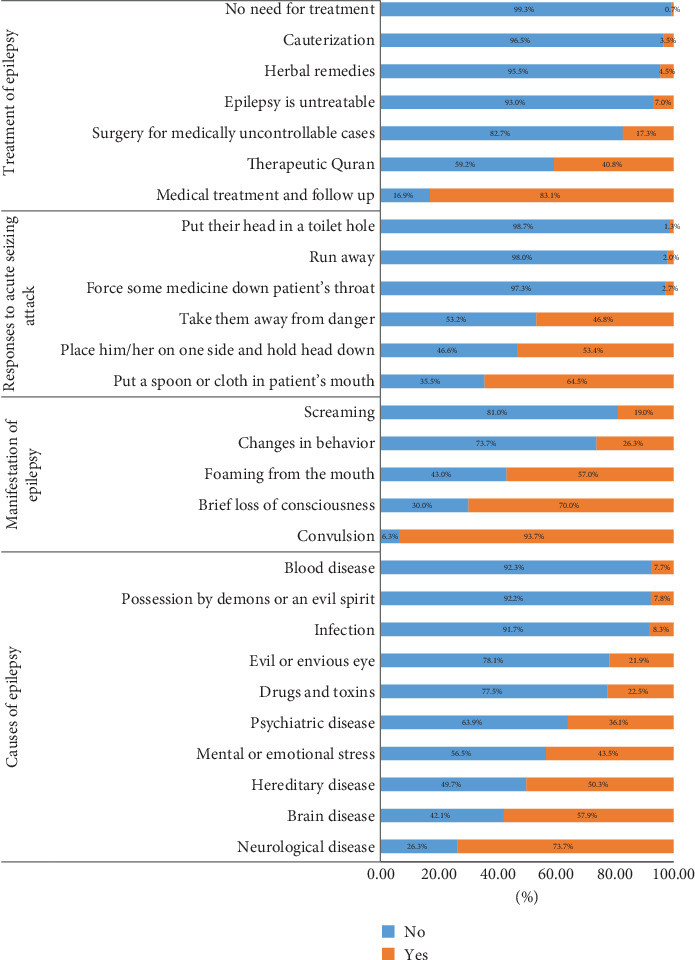
Distribution of respondents across knowledge about epilepsy.

Figure 2 depicts the responses to knowledge questions, indicating an acceptable level of understanding about epilepsy among the participants in the current study. When asked about the cause of epilepsy, 73.7% identified it as a neurological disease, 57.9% as a brain disease, and half of the respondents (50.3%) said it was a hereditary disease. However, 36% indicated they believe epilepsy is a psychiatric disease, with 21.9% stating it is caused by an “evil eye.”

The majority of responders (93.7%) indicated convulsions as a manifestation of epilepsy, whereas 26.3% thought it could manifest as a change in behavior. In terms of immediate management of acute seizing episodes, 64.5% mentioned putting a spoon or cloth in the patient's mouth, and 53.4% indicated they would place the patient on one side and hold their head down. Regarding the best treatment for epilepsy, 83.1% stated that medical treatment and follow-up were the best option. However, 40.8% mentioned reciting the Quran as an appropriate form of treatment, and 7% believed that epilepsy is untreatable. Additionally, 56.2% of participants were unaware that surgery could be a treatment option for medically uncontrollable epilepsy, and 26.5% expressed uncertainty about whether surgery is a viable option. The average knowledge score about epilepsy for the sample was 22 (SD ± 3.2), indicating that the participants had moderate knowledge about epilepsy.

**Figure 3:**
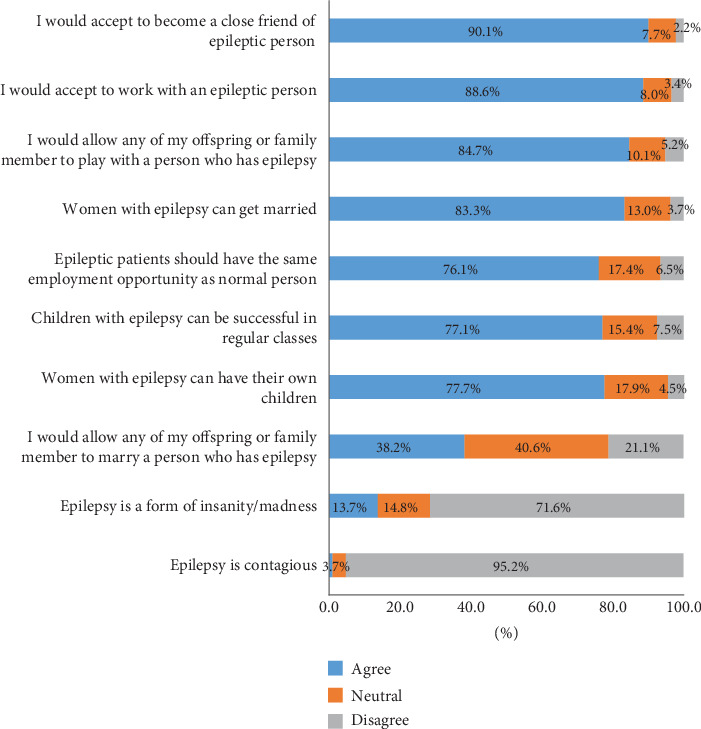
Distribution of respondents across attitude toward epilepsy.

The attitudes of the respondents toward epilepsy are depicted in Figure 3. Most respondents (95.2%) disagreed with the idea that epilepsy is contagious, and 71.6% disagreed with the claim that it is a form of insanity or madness. Additionally, 83% and 78% of individuals agreed that epileptic women can marry and have children, respectively. Similarly, 77.1% agreed that epileptic children can succeed in regular classes, and 76.1% agreed that epileptic persons should have equal employment opportunities. Further, 84.7% would permit their children to play with a peer who has epilepsy. Most participants expressed a willingness to work with PWE and form close friendships with them (88.6% and 90.1%, respectively). However, only 38.2% of respondents were accepting of the idea of their children or family members marrying someone with epilepsy. The mean attitude toward epilepsy was 27.14 (SD ± 3.1).

**Figure 4:**
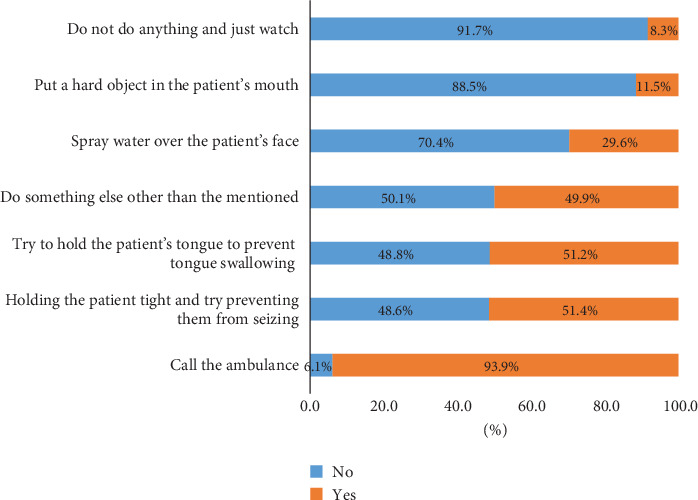
Distribution of respondents across actions toward seizure attacks.

Figure 4 shows responses to seizure attacks. The majority (93.9%) of participants indicated they would call an ambulance during a convulsive seizure. Additionally, 51.4% would hold the patient tightly, 51.2% would try to hold the patient's tongue, 29.6% would spray water over the patient's face, while 8.3% would choose to merely observe without intervening.

Females demonstrated significantly higher knowledge of epilepsy, a more positive attitude toward epilepsy, and a more appropriate response when witnessing a seizure compared to males (*p* < 0.001). Participants aged 18–30 exhibited good knowledge of epilepsy and a more appropriate response when witnessing a seizure compared to those in other age groups (*p* < 0.001). We noted significant differences in epilepsy knowledge and actions when witnessing a seizure based on marital status, occupation, and family income. Single individuals and students demonstrated better knowledge and responses during seizures (*p* = 0.002, *p* < 0.001, and *p* < 0.001, respectively). Participants with incomes less than 15,000 SR also demonstrated better knowledge (*p* = 0.001) and responses during seizures (*p* = 0.045) compared to those with higher incomes. Participants who had prior exposure to information about epilepsy, had a family member diagnosed with epilepsy, or had attended a course on controlling epileptic seizures displayed a higher level of knowledge about epilepsy (*p* < 0.001), a positive attitude toward epilepsy (*p* < 0.001), and better actions during seizure attacks (*p* < 0.001) (Tables 2, 3, and 4).

Pearson's correlation analysis was conducted to analyze the association between knowledge, attitude, and awareness about epilepsy. The results indicated that all variables were significantly and positively associated with each other (*p* < 0.001) (Table 5).

## 5. Discussion

The study found a high level of awareness about epilepsy in the Madinah community, as a significant proportion of respondents had heard or read about epilepsy (92.2%). However, only a small proportion of people had received formal education about epilepsy (12.8%), which significantly affected their understanding of the disease. They strongly believed that epilepsy is a form of psychiatric disease (36.1%) or mental and emotional stress (43.5%), showing their misconceptions about the disease. In addition, half of the respondents attributed epilepsy to hereditary factors (50.3%), which, although it may be accurate in certain instances, is not universally correct. Such beliefs also had a significant impact on people's attitudes, particularly regarding marrying someone with epilepsy. Moreover, a notable proportion associated seizures with possession by demons (7.8%) or an evil spirit (21.9%). This finding is consistent with previous studies conducted across various regions in Saudi Arabia, including Jeddah [11], Rabigh [16], Majmaah [19], Taif [20], and Al-Kharj [21] as well as in other Gulf countries like Yemen [23] and Iran [24]. Such beliefs may contribute to the societal stigma surrounding epilepsy. Addressing these misconceptions is crucial, as they play a pivotal role in fostering a more informed and empathetic community attitude toward PWE.

The study also found that a considerable percentage of respondents had witnessed someone having a seizure (43.1%). However, it also revealed certain misconceptions about appropriate first aid measures, such as placing a spoon or piece of cloth in the patient's mouth. Similar results were reported in studies conducted in Al-Majmaah [19], Rabigh [16], and Taif [20] in Saudi Arabia as well as in the United Kingdom [25] and Palestine [26]. Additionally, nearly half of the respondents said they would “try to hold the patient's tongue to prevent tongue swallowing” (51.2%) or “hold the patient tight and try preventing them from seizing” (51.4%). Furthermore, 29.6% of respondents said they would “spray water over the patient's face,” which is a higher percentage than reported in Jeddah [17] and Palestine [26]. This highlights a critical area for educational intervention to improve public response during seizures. Surprisingly, the participants also failed to recognize that surgery is a treatment option in refractory cases, reflecting a knowledge and awareness gap in medical treatment. This finding is similar to public awareness of surgery outcomes reported in Makkah City [14] and Palestine [26] but dissimilar to public awareness of surgical outcomes in western countries such as Canada [27], the United States [28], and Norway [29]. This suggests a strong need for targeted educational initiatives in Saudi Arabia to enhance awareness of available medical treatments, potentially bridging the gap between nonmedical beliefs and essential medical intervention.

In terms of attitudes toward PWE, the participants generally reported a positive attitude. For instance, a majority (95.2%) disagreed with the statement that “Epilepsy is contagious.” Additionally, positive attitudes were expressed regarding the reproductive rights of women with epilepsy and the potential success of children with epilepsy in regular classrooms. The participants also showed positive attitudes toward the employment of individuals with epilepsy, contrasting with findings from other regions [11, 16, 19–21].

The study also found several significant associations between sociodemographic characteristics and the participants' knowledge, attitudes, and actions. For example, females were found to be more knowledgeable and more likely to have positive attitudes and engage in positive actions in relation to epilepsy. Similar findings about sex differences were previously established in both local and international studies [20, 21, 25, 26, 30–35]. However, these findings contrast with those published in other studies, which found negative attitudes to be more associated with the female sex [22, 36]. Although a study conducted in Taif found that male individuals have stigmatizing and negative attitudes [20], one study in Qassim and one in the Eastern Province of Saudi Arabia found that males reported having higher levels of knowledge [13, 15]. Another study conducted in the United Kingdom found more positive attitudes in men than in women [25]. While a study in Iran did not find any significant association between knowledge and gender, it did report a significant association between attitude and female gender [33]. On the contrary, a study conducted in Palestine revealed a significant association between gender and knowledge but no significant association between gender and attitude [26]. In a Moscow-based study, gender was only significantly associated with the understanding of epilepsy as a curable disease but not with attitude [34]. Previous research has also revealed gender-specific attitudes toward people living with epilepsy in Nigeria [35] and Bahrain [32]. The inconsistent findings regarding sex and its association with knowledge and attitudes toward epilepsy might indicate that the effect of sex may be influenced by a multitude of factors.

In this study, younger people aged 18–30 years were more knowledgeable about epilepsy than older adults. This finding is consistent with studies conducted in Qassim [13], the Eastern Province of Saudi Arabia [15], the rural Cameroon Region in Central Africa [36], Ethiopia [28], and Norway [29], which also concluded that younger age is associated with better knowledge of epilepsy. One possible explanation for this trend could be the increased accessibility and exposure to information through modern educational resources, including the Internet and educational campaigns, which are more commonly utilized by members of the younger age group. However, age was not found to be significantly associated with knowledge among school teachers in Bahrain [32] and Iran [24, 33]. Moreover, age was not significantly associated with attitudes toward epilepsy in a study conducted in Moscow [34]. This insignificant association could be attributed to the fact that people of all ages in these countries are susceptible to developing epilepsy, causing them to become more knowledgeable and have positive attitudes toward it.

A higher level of education is another factor that was found to positively influence the level of knowledge and attitudes of the residents of Madinah Al-Munawara toward epilepsy. This finding is consistent with several published studies in different regions [20–23, 26, 28, 33, 34, 36–38]. However, previous studies reported no significant association between education and knowledge among school teachers in Bahrain [32] as well as between education and attitudes toward epilepsy in the general UK population [25]. Nevertheless, it is evident that in most of the cases, education is associated with a high level of knowledge and positive attitudes toward epilepsy, which highlights the importance of educational initiatives to spread awareness and create a positive understanding of this disease.

In this study, people who had family members with epilepsy and those who had attended a course on controlling seizures had better knowledge, took more positive actions, and had higher attitude scores. Similar results were reported in the populations of Al-Kharj [21], Cameroon [36], the Eastern Province of Saudi Arabia [15], the United Kingdom [25], Palestine [26], and Moscow [34]. However, economic status was not found to be significantly associated with knowledge among school teachers in Bahrain [32] and Iran [33]. Based on these findings, educational programs are needed to increase awareness about epilepsy and emphasize positive attitudes and actions toward it.

Surprisingly, in our study, people with an income of less than 15,000 SR were found to be more knowledgeable and take more action in comparison to those with a higher income. This contradicts the results of four studies that correlated higher income with more knowledge and positive attitudes and vice versa [16, 23, 31, 35]. A possible explanation for this unexpected finding could be that individuals with lower incomes might be more motivated to seek information and education about epilepsy due to a perceived need for self-reliance in managing their health concerns. Additionally, lower-income individuals might have more exposure to educational programs or community outreach initiatives that focus on health issues, including epilepsy. It is crucial to explore the specific factors influencing knowledge acquisition and positive actions in different income groups through further research and targeted investigations.

## 6. Strengths and Limitations

The study contributes to the literature by associating demographic characteristics with knowledge, awareness, attitudes, and actions taken in relation to epilepsy. It critically explored how specific factors influence knowledge acquisition and actions to combat epilepsy as well as how awareness translates into people's attitudes and actions in relation to epilepsy. Further, the study highlighted the impact of spreading information on epilepsy on public knowledge obtained through educational programs. It also pinpointed the demographic groups that are more likely to have good knowledge, positive attitudes, high awareness, and good action plans in relation to epilepsy. The findings of this study can be used to focus on demographic groups that lack knowledge and awareness and have bad attitudes toward epilepsy to help mitigate the negative impact of this disease on society.

This study is aimed at evaluating the awareness, knowledge level, and attitudes toward seizure attacks of the general population of Madinah City. Every attempt was made to obtain precise, reliable, and satisfactory results. Nonetheless, some limitations must be acknowledged. First, an online questionnaire was used for the study. A physical form could have yielded more accurate results given that older adults may not have the computer skills to access such a survey, and part of the population that may have lacked reliable internet service could not be reached. Second, since the survey was distributed by 50 volunteers, family and friends with similar attitudes may have been recruited. As a result, further research on this subject is needed using various study designs and sociodemographic data. Third, the study only focused on knowledge, attitudes, and awareness about epilepsy without considering the experiences and general practices of PWE. Future research should focus on how experiences and general practices related to epilepsy are shaped and may affect individuals' knowledge, attitudes, and awareness about epilepsy.

## 7. Conclusion

Our results showed that the general public's attitudes toward epilepsy were reasonably favorable and their knowledge levels were acceptable. However, the data also revealed that the respondents had some misconceptions, such as the incorrect belief that epilepsy is a psychological disorder or that it is caused by an “evil eye.” Additionally, most of the participants were unaware that surgery is a treatment option for refractory cases, and the majority lacked knowledge of appropriate actions to take when witnessing a seizure. Consequently, educational awareness initiatives are needed to increase public understanding of epilepsy and appropriate actions to take during a seizure. This could help to reduce stigma and better support PWE. The results of this study can be used to support global comparisons of epilepsy with the situation in Saudi Arabia.

## Figures and Tables

**Table 1:** Sociodemographic characteristics of the sample (*n* = 2626).

**Characteristics**	**Frequency**	**Percentages**
*Gender*		
Male	864	32.9%
Female	1762	67. 1%
*Age groups in years*		
18–30	1724	65.7%
31–49	695	26.5%
50 or more	207	7.9%
*Marital status*		
Single	1674	63.7%
Married	925	35.2%
Divorced	19	0.7%
Widowed	8	0.3%
*Educational level*		
Uneducated	2	0.1%
Primary school	11	0.4%
Intermediate school	18	0.7%
High school	761	29%
Diploma	75	2.9%
Bachelor	1623	61.8%
Postgraduate	136	5.2%
*Occupation*		
Student	1322	50.3%
Employee	788	30.0%
Self-employed	60	2.3%
Retired	85	3.2%
Housewife	206	7.8%
Unemployed	165	6.3%
*Family monthly income (SR)*		
Less than 5000	809	30.8%
5001–10,000	644	24.5%
10,000–15,000	478	18.2%
15,000–20,000	387	14.7%
More than 20,000	308	11.7%

**Table 2:** Association between sociodemographic characteristics and knowledge about epilepsy (*n* = 2626).

**Characteristics**	**Categories**	**Knowledge**		**p** ** value**
		**Poor** **(** **n** = 1115**)**	**Good** **(** **n** = 1511**)**	
Gender	Male	445 (39.9%)	419 (27.7%)	< 0.001⁣^∗^
	Female	670 (60.1%)	1092 (72.3%)	

Age group in years	18–30	684 (61.3%)	1040 (68.8%)	< 0.001⁣^∗^
	31–49	329 (29.5%)	366 (24.2%)	
	50 or more	102 (9.1%)	105 (6.9%)	

Marital status^a^	Single	665 (59.6%)	1009 (66.8%)	0.002⁣^∗^
	Married	436 (39.1%)	489 (32.4%)	
	Divorced	10 (0.9%)	9 (0.6%)	
	Widowed	4 (0.4%)	4 (0.3%)	

Educational level^a^	Uneducated	1 (0.1%)	1 (0.1%)	0.013⁣^∗^
	Primary school	9 (0.8%)	2 (0.1%)	
	Intermediate school	9 (0.8%)	9 (0.6%)	
	High school	326 (29.2%)	435 (28.8%)	
	Diploma	43 (3.9%)	32 (2.1%)	
	Bachelor	670 (60.1%)	953 (63.1%)	
	Postgraduate	57 (5. 1%)	79 (5.2%)	

Occupation	Student	502 (45.0%)	820 (54.3%)	< 0.001⁣^∗^
	Employee	365 (32.7%)	423 (28.0%)	
	Self-employee	33 (3.0%)	27 (1.8%)	
	Retired	43 (3.9%)	42 (2.8%)	
	Housewife	95 (8.5%)	111 (7.3%)	
	Unemployed	77 (6.9%)	88 (5.8%)	

Family monthly income (SR)	Less than 5000	361 (32.4%)	448 (29.6%)	0.001⁣^∗^
	5001–10,000	273 (24.5%)	371 (24.6%)	
	10,000–15,000	220 (19.7%)	258 (17.1%)	
	15,000–20,000	163 (14.6%)	224 (14.8%)	
	More than 20,000	98 (8.8%)	210 (13.9%)	

Have you ever heard or read about epilepsy?	No	154 (13.8%)	52 (3.4%)	< 0.001⁣^∗^
	Yes	961 (86.2%)	1459 (96.6%)	

Do you have a family member diagnosed with epilepsy?	No	959 (86.0%)	1185 (78.4%)	< 0.001⁣^∗^
	Yes	156 (14.0%)	326 (21.6%)	

Have you ever attended a course on controlling an epileptic seizure?	No	1031 (92.5%)	1260 (83.4%)	< 0.001⁣^∗^
	Yes	84 (7.5%)	251 (16.6%)	

^a^Fisher's exact test.

⁣^∗^*p* value is statistically significant.

**Table 3:** Association between sociodemographic characteristics and attitude toward epilepsy (*n* = 2626).

**Characteristics**	**Categories**	**Attitude**		**p** ** value**
		**Negative** **(** **n** = 1038**)**	**Positive** **(** **n** = 1588**)**	
Gender	Male	429 (41.3%)	435 (27.4%)	< 0.001⁣^∗^
	Female	609 (58.7%)	1153 (72.6%)	

Age group in years	18–30	667 (64.3%)	1057 (66.6%)	0.245
	31–49	293 (28.2%)	402 (25.3%)	
	50 or more	78 (7.5%)	129 (8.1%)	

Marital status^a^	Single	654 (63.0%)	1020 (64.2%)	0.647
	Married	371 (35.7%)	554 (34.9%)	
	Divorced	10 (1.0%)	9 (0.6%)	
	Widowed	3 (0.3%)	5 (0.3%)	

Educational level^a^	Uneducated	1 (0.1%)	1 (0.1%)	0.910
	Primary school	5 (0.5%)	6 (0.4%)	
	Intermediate school	9 (0.9%)	9 (0.6%)	
	High school	298 (28.7%)	463 (29.2%)	
	Diploma	29 (2.8%)	46 (2.9%)	
	Bachelor	647 (62.3%)	976 (61.5%)	
	Postgraduate	49 (4.7%)	87 (5.5%)	

Occupation	Student	518 (49.9%)	804 (50.6%)	0.201
	Employee	320 (30.8%)	468 (29.5%)	
	Self-employee	31 (3.0%)	29 (1.8%)	
	Retired	37 (3.6%)	48 (3.0%)	
	Housewife	75 (7.2%)	131 (8.2%)	
	Unemployed	57 (5.5%)	108 (6.8%)	

Family monthly income (SR)	Less than 5000	342 (32.9%)	467 (29.4%)	0.078
	5001–10,000	263 (25.3%)	381 (24.0%)	
	10,000–15,000	178 (17.1%)	300 (18.9%)	
	15,000–20,000	151 (14.5%)	236 (14.9%)	
	More than 20,000	104 (10.0%)	204 (12.8%)	

Have you ever heard or read about epilepsy?	No	128 (12.3%)	78 (4.9%)	< 0.001⁣^∗^
	Yes	910 (87.7%)	1510 (95.1%)	

Do you have a family member diagnosed with epilepsy?	No	890 (85.7%)	1254 (79.0%)	< 0.001⁣^∗^
	Yes	148 (14.3%)	334 (21.0%)	

Have you ever attended a course on controlling an epileptic seizure?	No	944 (90.9%)	1347 (84.8%)	< 0.001⁣^∗^
	Yes	94 (9.1%)	241 (15.2%)	

^a^Fisher's exact test.

⁣^∗^*p* value is statistically significant.

**Table 4:** Association between sociodemographic characteristics and action toward seizure attacks (*n* = 2626).

**Characteristics**		**Actions**		**p** ** value**
	**Categories**	**Bad** **(** **n** = 1190**)**	**Good** **(** **n** = 1436**)**	
Gender	Male	431 (36.2%)	433 (30.2%)	< 0.001⁣^∗^
	Female	759 (63.8%)	1003 (69.8%)	

Age group in years	18–30	715 (60.1%)	1009 (70.3%)	< 0.001⁣^∗^
	31–49	367 (30.8%)	328 (22.8%)	
	50 or more	108 (9.1%)	99 (6.9%)	

Marital status^a^	Single	694 (58.3%)	980 (68.2%)	< 0.001⁣^∗^
	Married	484 (40.7%)	441 (30.7%)	
	Divorced	8 (0.7%)	11 (0.8%)	
	Widowed	4 (0.3%)	4 (0.3%)	

Educational level	Uneducated	2 (0.2%)	0 (0.0%)	0.059
	Primary school	8 (0.7%)	3 (0.2%)	
	Intermediate school	11 (0.9%)	7 (0.5%)	
	High school	337 (28.3%)	424 (29.5%)	
	Diploma	40 (3.4%)	35 (2.4%)	
	Bachelor	739 (62.1%)	884 (61.6%)	
	Postgraduate	53 (4.5%)	83 (5.8%)	

Occupation	Student	536 (45.0%)	786 (54.7%)	< 0.001⁣^∗^
	Employee	386 (32.4%)	402 (28.0%)	
	Self-employee	34 (2.9%)	26 (1.8%)	
	Retired	43 (3.6%)	42 (2.9%)	
	Housewife	112 (9.4%)	94 (6.5%)	
	Unemployed	79 (6.6%)	86 (6.0%)	

Family monthly income (SR)	Less than 5000	381 (32.0%)	428 (29.8%)	0.045⁣^∗^
	5001–10,000	287 (24.1%)	357 (24.9%)	
	10,000–15,000	236 (19.8%)	242 (16.9%)	
	15,000–20,000	164 (13.8%)	223 (15.5%)	
	More than 20,000	122 (10.3%)	186 (13.0%)	

Have you ever heard or read about epilepsy?	No	124 (10.4%)	82 (5.7%)	< 0.001⁣^∗^
	Yes	1066 (89.6%)	1354 (94.3%)	

Do you have a family member diagnosed with epilepsy?	No	992 (83.4%)	1152 (80.2%)	0.039⁣^∗^
	Yes	198 (16.6%)	284 (19.8%)	

Have you ever attended a course on controlling an epileptic seizure?	No	1099 (92.4%)	1192 (83.0%)	< 0.001⁣^∗^
	Yes	91 (7.6%)	244 (17.0%)	

^a^Fisher's exact test.

⁣^∗^*p* value is statistically significant.

**Table 5:** Pearson's correlation analysis—association between knowledge about epilepsy, attitude toward epilepsy, and awareness about epilepsy (*n* = 2626).

		**M**	**SD**	**1**	**2**	**3**
1	Knowledge about epilepsy	22.0	3.18	1.00		
2	Attitude toward epilepsy	27.1	3.07	0.318⁣^∗∗∗^	1.00	
3	Awareness about epilepsy	1.82	0.71	0.342⁣^∗∗∗^	0.205⁣^∗∗∗^	1.00

⁣^∗∗∗^*p* < 0.001.

## Data Availability

Data are available upon request from the corresponding author.
